# Dynamic Transcriptional Landscape of the Early Chick Embryo

**DOI:** 10.3389/fcell.2019.00196

**Published:** 2019-09-12

**Authors:** Junxiao Ren, Congjiao Sun, Michael Clinton, Ning Yang

**Affiliations:** ^1^Department of Animal Genetics and Breeding, College of Animal Science and Technology, China Agricultural University, Beijing, China; ^2^National Engineering Laboratory for Animal Breeding, Key Laboratory of Animal Genetics, Breeding and Reproduction, Ministry of Agriculture and Rural Affairs, China Agricultural University, Beijing, China; ^3^Division of Developmental Biology, The Roslin Institute and Royal (Dick) School of Veterinary Studies, University of Edinburgh, Midlothian, United Kingdom

**Keywords:** embryonic development, RNA-Seq, Iso-Seq, intron retention, chicken

## Abstract

Defining the dynamic transcriptome of the early embryo at high resolution would assist greatly in understanding vertebrate development. Here, we describe the dynamic transcription landscape of early chick embryo development using advanced single-molecule long-read isoform sequencing (Iso-Seq) and RNA-Seq technology. Our transcriptomic profiling reflected the time course of chicken embryonic development from day 1 to day 8 of incubation, a period encompassing gastrulation, somitogenesis, and organogenesis. This analysis identified transcriptional isoforms, alternative splicing (AS) events, fusion transcripts, alternative polyadenylation (APA) sites, and novel genes. Our results showed that intron retention (IR) represented the most abundant AS type and displayed distinct features and dynamic modulation during development. Moreover, we constructed a high-resolution expression profile across embryonic development. Our combined expression dataset correlates distinct gene clusters with specific morphological changes, and provides the first framework for the molecular basis of early chicken embryogenesis. Analysis of gene expression in the developing chicken embryo highlighted the dynamic nature and complexity of the chicken transcriptome and demonstrated that dramatically increased IR events are associated with distinct gene sets.

## Introduction

The vertebrate embryo undergoes extensive morphological changes during the processes of gastrulation and organogenesis (Mitiku and Baker, 2007). Intricate spatiotemporal control of gene expression is undoubtedly critical to these morphological changes. Therefore, defining the dynamic expression of the transcriptome over time would be an important step toward understanding the regulatory processes governing embryonic development. The dynamic transcriptional landscape of the human embryo (Fang et al., 2010) and of many of the most popular model animal embryos have been well-characterized in recent years, including mouse (Mitiku and Baker, 2007), zebrafish (Nepal et al., 2013; White et al., 2017), *Drosophila melanogaster* (Graveley et al., 2011), and nematode (Levin et al., 2012; West et al., 2018). A previous study of transcriptome profiles of human embryos during early development devised a putative molecular network that may provide a framework for the regulation of early human organogenesis (Fang et al., 2010). In zebrafish, researchers constructed a high-resolution transcriptional profile of embryonic development, and their results showed a burst of transcription of highly related zinc finger proteins during zygotic genome activation (White et al., 2017). Analysis of the dynamic transcriptome during mouse gastrulation and organogenesis defined groups of genes that have distinct functions during development (Mitiku and Baker, 2007). These data represent a powerful resource for studying developmental gene regulation and reveal the functional potential of patterned genes during embryonic development.

Of the post-transcriptional mechanisms proposed to increase transcriptome complexity, alternative splicing (AS) and alternative polyadenylation (APA) are considered the most widespread (Pan et al., 2008; Ozsolak et al., 2010; Braunschweig et al., 2014; Brown et al., 2014). As a result of AS, many multi-exon genes produce multiple transcript isoforms, resulting in the transcriptomic complexity (Pan et al., 2008). An example of the potential complexity that can arise from AS exists with the *Drosophila* homolog of a human Down syndrome cell adhesion molecule gene, which can generate more than 38,000 isoforms by AS (Schmucker et al., 2000). Intron retention (IR), which is the process that occurs when a specific intron(s) remains unspliced in the mature polyadenylated transcripts, is one of the most widespread AS types (Braunschweig et al., 2014; Ni et al., 2016; Pimentel et al., 2016; Naro et al., 2017). Widespread IR is emerging as a mechanism for gene regulation during differentiation and development (Braunschweig et al., 2014; Jung et al., 2015). Recently, IR was reported to strongly programed and therefore regulate the terminal erythropoiesis (Pimentel et al., 2016), CD4^+^ T cell activation (Ni et al., 2016), germ cell differentiation (Naro et al., 2017), and granulocyte differentiation (Wong et al., 2013).

RNA-Seq technology has been widely applied to detect gene expression and AS events (Pan et al., 2008; Sultan et al., 2008). However, it is still technically challenging to accurately reconstructing full-length splice variants using short sequencing reads (Abdel-Ghany et al., 2016). Single-molecule long-read sequencing (Iso-Seq) technology has enabled a more accurate identification of spliced isoforms by sequencing full-length transcripts directly (Abdel-Ghany et al., 2016; Bo et al., 2016; Kuo et al., 2017). Iso-Seq has shown that the chicken transcriptome is of similar complexity to that of humans (Kuo et al., 2017). However, the extent to which AS events contribute to transcriptome complexity and how these events drive embryonic development are largely unknown.

For many reasons, especially the rapid *in ovo* development, the chicken embryo is a valuable vertebrate model system for embryological studies and medical research (Brown et al., 2003; Stern, 2005). The rapid development combined with a high-quality genome makes the chicken embryo an excellent system for studying transcriptome dynamics. The morphological changes that occur during chicken embryogenesis have been well-documented (Hamburger and Hamilton, 1951; Kochav et al., 1980; Watt et al., 1993). According to the description of morphology, embryos at day 1–8 of incubation (labeled E1–E8; represent the Hamburger-Hamilton stage 6–34) encompass entire gastrulation, somitogenesis, and organogenesis, as well as major morphological changes (formation of limb and craniofacial etc.). For the first 24 h of incubation, the primitive streak appears and then reaches its maximum length. A rapidly increasing in number of somites occurs in the next 24 h incubation. Then the numbers of somites become increasingly difficult and organ structures (Limbs, Visceral arches and Allantois etc.) become visible. After about 6 days of incubation, many organs and structures begin to acquire the morphology typical of chicken species. However, little is known of the intricate molecular changes during these processes. Our objective is to supplement the well-established morphological data by characterizing global AS events and gene expression profiles during chicken embryo development.

## Materials and Methods

### Ethics Statement

All animal experiments were approved by the Animal Welfare Committee of China Agricultural University (permit number XK622) and performed in accordance with the protocol outlined in the “Guide for Care and Use of Laboratory Animals” (China Agricultural University).

### Sample Collection

Fertilized eggs from a pure line of White Leghorns were collected and used in this study. The eggs were disinfected and incubated in an automated egg incubator at 37.5°C and 65% relative humidity, with rotation every 6 h. Embryos were collected at 24 h intervals and three biological replicates were generated for each developmental stage, labeled E1–E8. Each biological replicate from the E1, E2, and E3 groups comprised six, four, and three embryos, respectively. Additionally, the three biological replicates for the remaining developmental stages (E4–E8) comprised three individual embryos each. Embryos were rinsed in 1 × PBS and immediately processed for RNA isolation. Total RNA was extracted with Trizol reagent following the manufacturer's instructions. The RNA concentration and purity were measured using the NanoDrop 2000 spectrophotometer (Thermo Fisher Scientific, Wilmington, DE, USA). The integrity of the RNA was determined using an Agilent 2100 Bioanalyzer (Agilent Technologies, CA, USA).

### Illumina Library Construction and Sequencing

Equal amounts of RNA (1.5 μg) per sample were used to prepare the Ribo-Zero RNA-sequencing library. Briefly, rRNA was removed using the Ribo-Zero rRNA Removal Kit (Epicentre, Madison, WI, USA). Sequencing libraries were generated using NEBNext^R^ Ultra™ Directional RNA Library Prep Kit for IlluminaR (New England Biolabs, Ipswich, MA, USA). The set of 24 prepared libraries were sequenced with the Illumina HiSeq 4000 platform using the 150-bp pair-end sequencing strategy, following the manufacturer's instructions.

### PacBio Library Construction and Sequencing

RNA samples extracted from E1, E3, E5, and E7 embryos were used to prepare the PacBio library. For each developmental stage, equal amounts of total RNA from three biological replicates were pooled together. High-quality RNA samples were used to construct the libraries. Full-length cDNA was produced using the SMARTer™ PCR cDNA Synthesis Kit. The cDNA was then amplified by 16 cycles of PCR and then size selected using BluePippin (Sage Science, Beverly, MA, USA) into the following bins for each sample: 1–2, 2–3, and 3–6 kb. These three libraries for each sample were generated using SMRTbell Template Prep Kit 1.0 (Pacific Biosciences, Menlo Park, CA, USA). The 12 libraries were sequenced on the Pacific Bioscience RS II platform with 13 SMRT cells. Subsequently, for each developmental stage, equal amounts of different lengths of cDNA (1–2, 2–3, and 3–6 kb) were pooled together to generate a full-length (0–6 kb) cDNA library. The resulting four full-length libraries from E1, E3, E5, and E7 were then sequenced on a PacBio Sequel platform using C3 reagents with four SMRT cells.

### PacBio Long-Read Processing

PacBio data were processed and evaluated using the PacBio SMRT Analysis Package (v2.3.0; http://www.pacificbiosciences.com) with default parameters. Briefly, raw reads in fastq format were extracted by pbh5tools packages (v0.8.0). The obtained reads were further processed into error corrected reads of insert with a minimum filtering requirement of 0 and a minimum read quality of 75%. Next, reads of inserts that contained 5′ and 3′ adapters used in the library preparation as well as the poly(A) tail in the expected arrangement are classified as full-length non-chimeric reads (FLNC). Iterative Clustering for Error Correction was used to obtain consensus isoforms. Further nucleotide errors in consensus isoforms were corrected using the Illumina RNA-Seq short reads. The error-corrected FLNC sequences were then mapped to the Galgal 5 reference genome assembly using GMAP (Wu and Watanabe, 2005). Mapped reads were further collapsed using the pbtranscript-ToFU package with min-coverage = 85% and min-identity = 90%. The 5′ difference was not considered when collapsing redundant transcripts. The collapsed transcripts from all 16 PacBio libraries were then merged using in-house Python scripts to create a PacBio GTF file.

AS events were detected using SpliceGrapher (v0.2.6; Rogers et al., 2012) based on the above GTF file. Five major types of AS events, namely, IR, Exon skipping (ES), Alternative 5′ splicing site (A5SS), Alternative 3′ splicing site (A3SS), and Mutually exclusive exon (MEE), were extracted from the output files and counted. Polyadenylation analysis was carried out using all FLNC reads. To address the possibility of multiple reads occurring at one poly(A) site and to ignore microheterogeneity of APA, a short sequence of 20 nt was used to cluster microheterogeneity sites according to the default option of TAPIS (Abdel-Ghany et al., 2016). To identify the poly(A) signal, MEME was employed for motif searches on the 50-nucleotide sequences upstream of the polyadenylation sites (Bailey et al., 2009). TopHat2 (v2.0.12) was employed to identify fusion transcripts (Kim et al., 2013). The criteria for fusion candidates were as follows: (a) a single transcript must map to two or more loci; (b) loci on contigs or scaffolds are not acceptable; (c) minimum coverage for each locus is 5%; (d) total coverage must be at least 95%; (e) distance between the loci is at least 10 kb; and (f) junction spanning sites must be supported by Illumina short reads.

### Premature Termination Codon Prediction

The longest representative RefSeq transcript was selected for each gene. The IR-containing transcripts of these genes were then subjected to predict the open reading frame using TransDecoder (Haas et al., 2013). Five hundred and seventy eight IR-containing transcripts were excluded from further premature termination codon analysis because the retained intron was outside of, or overlapping with, the coding sequence of the representative transcript. The longest open reading frame was selected and the corresponding protein sequence was BLAST with the Swissprot database. If the distance between the predicted stop codon and the last exon-exon junction was greater than 50 nucleotides, the stop codon was defined as a premature termination codon (Nagy and Maquat, 1998; Jung et al., 2015).

### Illumina Data Processing

Raw reads were cleaned using the FASTX-Toolkit (v0.0.13; http://hannonlab.cshl.edu/fastx_toolkit/). Clean reads with high quality were mapped to the reference genome of chicken (Galgal 5) using TopHat2 (v2.0.12). Only reads with a perfect match or one mismatch were employed to assemble transcripts using String Tie under the default parameters (Pertea et al., 2015). The assembled transcripts were annotated using the gffcompare program. Transcript expression levels were estimated by the FPKM method using the PacBio GTF annotation file.

### Reverse Transcription-Polymerase Chain Reaction (RT–PCR)

Total RNA was extracted as described above. For cDNA synthesis, 1 μg of RNA from each sample was treated with DNase I (Invitrogen, Carlsbad, CA #18068015) to remove trace amounts of DNA according to the manufacturer's instructions. The DNase I-treated RNA was then reverse transcribed into cDNA using the PrimeScript™ RT Reagent Kit (TaKaRa, Dalian, China # RR047A). PCR conditions were 95°C for 5 min, 35 cycles of 95°C for 30 s, 58°C for 30 s and 72°C for 30 s, followed by a further 10 min extension at 72°C. PCR amplification was monitored on 2% agarose gels. All primer sequences used were shown in Table S1.

## Results

### Complexity of the Chicken Transcriptome

**Figure 1:**
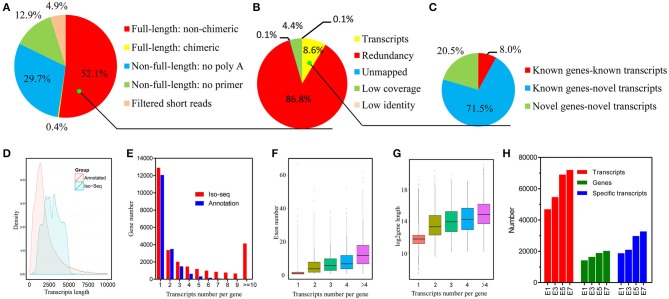
Identification and characterization of chicken embryo transcripts. **(A)** Raw Iso-Seq reads were divided into five groups to filter FLNC reads. **(B)** FLNC reads were divided into five groups based on their genome mapping characteristics to filter transcripts. **(C)** All transcripts were divided into three groups based on their genome annotations. **(D)** Density plot of the length of the PacBio transcripts and previously annotated transcripts. **(E)** Distribution of the number of transcripts per gene generated by PacBio data and reference genome data. **(F)** Comparison of exon numbers among genes producing different numbers of transcripts. **(G)** Comparison of gene length among genes producing different numbers of transcripts. **(H)** Identified genes and transcripts in embryos at different developmental stages by Iso-Seq.

In order to accurately identify high-quality transcripts in the developing chicken embryo, a total of 16 size-fractionated libraries (see Materials and Methods) were sequenced on the PacBio RS II and Sequel platform. In total, we obtained 3,012,330 reads of inserts, of which 1,570,107 (52.1%) were identified as FLNC reads (Figure 1A, Figure S1 and details in Table S2). All of the FLNC reads were polished using the algorithm of iterative clustering, and further error-corrected by non-FLNC reads and Illumina HiSeq 2000 short reads to construct consensus isoforms. The consensus isoforms were then mapped against the chicken genome by GMAP, yielding 135,379 high-quality transcripts (Figure 1B). The density plot of transcripts showed that the length of the identified PacBio transcripts was much longer than that of the annotated transcripts (Figure 1D). All of the 135,379 transcripts were classified into three groups based on the mapping results (Figure 1C). Of this total number, 107,401 (79.3%) transcripts aligned to 13,405 annotated genes, and of these, 10,820 transcripts had been previously described and 96,581 (71.3%) were newly discovered in this study. The remaining 27,627 transcripts that were not aligned to annotated genes, mapped to 14,955 novel loci. Consequently, we discovered a total of 124,559 new transcripts by Iso-Seq.

Among the 28,360 genes detected in the PacBio data, 12,896 genes (45.5%) were represented by a single transcript, indicating that approximately half of the genes were spliced (Figure 1E). As might be expected, we found that genes associated with more transcripts have more exons and are longer (Figures 1F,G). The number of transcripts detected rises steadily during the process of embryo development and a wider range of genes are expressed at later stages (Figure 1H). In addition, by using the filtered FLNC transcripts, we identified 1,333 fusion transcripts (see the Materials and Methods; Figure S1). The veracity of fusion events is strongly supported by the fact that 90% of the fusion junction sites occurred in at least one Illumina short read. Broadly speaking, these findings indicate that the complexity of the chicken transcriptome has been significantly underestimated.

### IR Events Dramatically Increase During Embryonic Development

**Figure 2:**
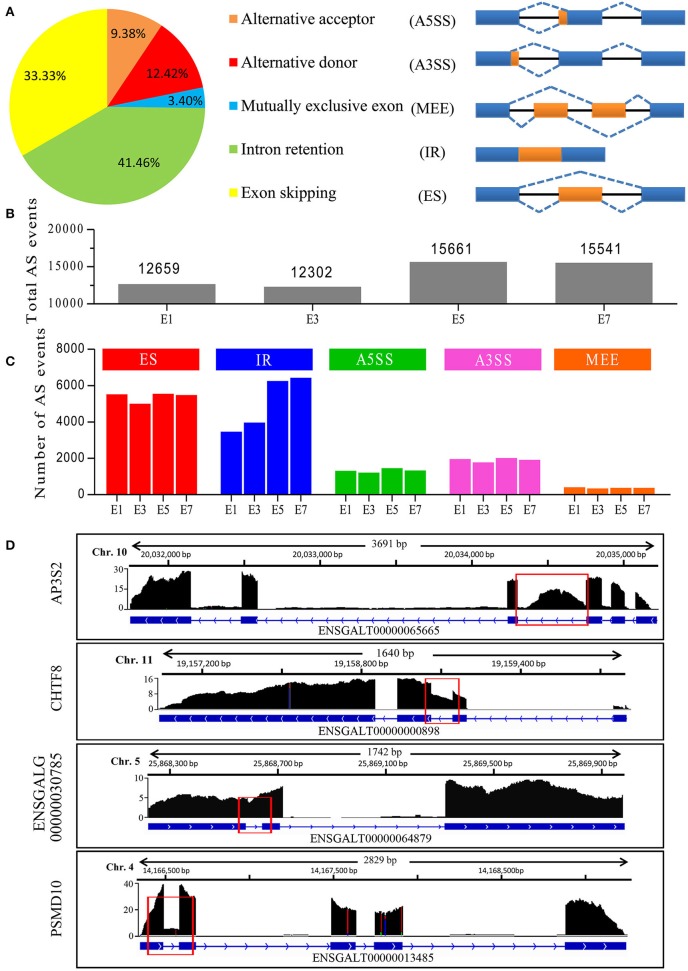
Characterization of AS events in chicken embryos identified by Iso-Seq. **(A)** Distribution of different types of AS events generated by all PacBio transcripts and a schematic illustrating the five AS modes. **(B)** Total AS events identified at different stages of chicken embryo development. **(C)** Number of different AS events at the different stages of embryo development. **(D)** Wiggle plots showing RNA-seq reads mapped to genes with significant IR events. Y-axis represented the normalization value (Reads per 10 million). The representative retained introns are marked with red rectangles.

Our Iso-Seq analysis identified 48,351 AS events, and they were empirically classified into five major AS types (Figure 2A). The majority of AS events were comprised of IR and ES events, with IR being the most abundant overall. Of these, only 393 AS events (<1%) were not supported by Illumina short reads. Nine AS events were selected and their validity was confirmed by RT-PCR analysis (Figure S2), demonstrating the accuracy of long reads in detecting AS events. We next delineated stage-associated AS events based on development at E1, E3, E5, and E7. An increase in the number of total AS events was observed between E3 and E5 (Figure 2B). Among all samples, the majority of AS events (>60%) are found across all developmental stages. In accordance with the analysis of transcripts, ~40% transcripts tend to be found at specific developmental stages (Figure 1H). For instance, we found that no *Blimp* isoforms were identified in sample E1 and only one isoform ENSGALT00000093326 was identified in E3. However, more than 5 isoforms were found in E5 and E7 sample. It's reported that Blimp1, which played a crucial roles in embryonic development (Ha and Riddle, 2003), was first detected in E2 chicken embryo, and then expressed in various embryonic tissues, indicating that these AS events were orchestrated rather than random. Interestingly, IR events gradually increased by 86% during embryonic development (Figure 2C), unlike other AS types. In particular, we totally identified 20,046 IR-containing transcripts in all samples. However, 7,904 (33.4%) transcripts were not detected at all four stages, tending to be stage-specific transcripts. Together, our results highlighted the developmental regulation of AS events, especially IR events, during embryonic development. It has been proposed that AS is a key mechanism underlying transcriptomic complexity (Brown et al., 2014), and the increase in IR observed during organogenesis suggests that IR may be a key mechanism driving this process during embryonic development.

We next determined whether IR events were caused by immature RNA transcripts. Preliminary inspection of RNA-Seq data, displaying that the majority of clean reads were mapped to gene exons (>80%), revealed that most introns were efficiently spliced. Analysis of the 20,046 IR-containing transcripts, 15,995 (79.8%) transcripts have only one retained intron, 3,540 (17.6%) transcripts have two retained introns and <3% transcripts have more than 2 retained introns. In contrast, 20,038 (99.9%) transcripts have spliced out more than one intron and 15810 (78.9%) transcripts have spliced out more than five introns, indicating that most introns were spliced out. If there are a large number of pre-mRNAs, there should be an appreciable quantity of transcripts retained multi-introns, indicating that these transcripts (introns) were stably existing, but not transient. For example, four genes in Figure 2D exhibited major peaks in read density over the exons and deep troughs in intronic regions, only one intron for each gene exhibited high peak in read density, revealing that the introns have been efficiently spliced out during pre-mRNA splicing. A prominent IR event was found in the chromosome transmission fidelity factor 8 (CHTF8), which encodes a protein that is essential for the fidelity of chromosome transmission. CHTF8 intron 2 was highly retained, while introns 1 and 3 were totally spliced out (Figure 2D). These results confirmed that these polyadenylated intron-retaining transcripts were bona fide functional transcripts.

### Dynamic IR Modulates the Expression of Functional Gene Classes

Further analysis of a set of 11,742 IR-containing transcripts that were generated by known protein coding genes identified in our data, we found that 11,164 (95.1%) IR events generated a premature termination codon. It has been reported that IR events often introduce premature termination codons, and lead to reduced mRNA and protein levels by triggering the non-sense-mediated decay mechanism (Nagy and Maquat, 1998; Jung et al., 2015). Additional analysis found that, of the 11,164 IR-containing transcripts, 36% (4,015) were predicted to have no complete open reading frame. BLAST analysis of the remaining 7,149 IR-containing transcripts, found that only 1,008 (14.1%) matched to known proteins (coverage > 50% and identity > 80%) in the Swissprot database, suggesting that most of these IR-containing transcripts have no protein coding potential. Taken together, these results suggest that IR events have a profound impact on gene expression.

**Figure 3:**
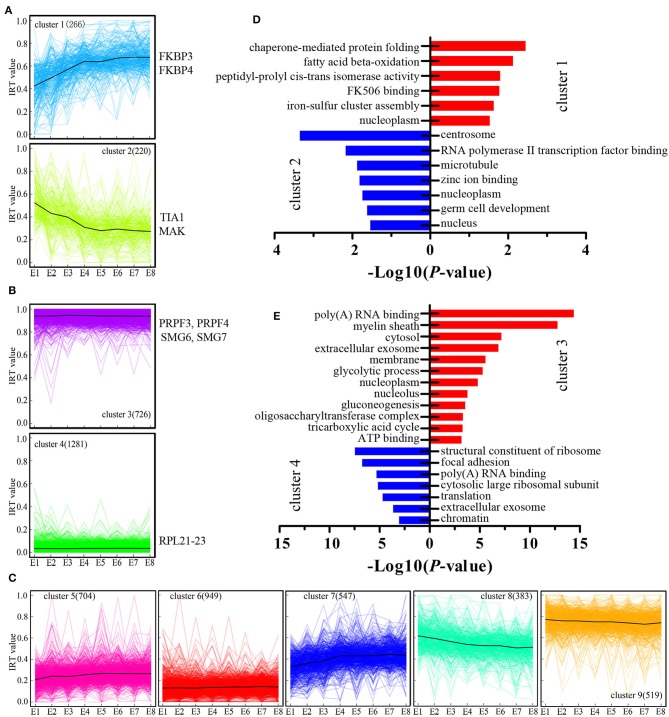
Cluster analysis of IR during chicken embryo development. **(A–C)** K-means cluster analysis based on IR-containing transcript values during the eight stages of embryo development. The number of IR-containing transcripts in each cluster is showed within parentheses. On the vertical axis, IRT value represents the IR-containing transcript values. Some representative known genes for each cluster were present near the cluster. **(D,E)** DAVID analysis of enriched GO terms for each gene cluster.

We next examined genes where their expression was modulated by IR events. We introduced the concept of “IR-containing transcript value” to better represent the changes associated with IR-containing transcripts. The IR-containing transcript value for each gene at each developmental stage was characterized by calculating the ratio of the sum of the FPKM value (generated by RNA-seq) of the IR-containing transcripts (numerator) to the sum of the FPKM value of all transcripts (denominator), resulting in IR-containing transcript values on a [0–1] scale. Next, all the genes were grouped into nine clusters based on their IR-containing transcript values at eight developmental stages using the K-means method (Figures 3A–C). For example, cluster C1 (Figure 3A), comprising 266 genes, represents the genes with developmentally increased IR-containing transcript levels. In other words, the genes in cluster 1 showed increasing regulation by IR events. Conversely, cluster C2 comprised 220 genes with developmentally decreased IR events. Cluster C3 and C4 constitute developmentally stable IR events with polarized IR-containing transcript values (Figure 3B). GO analysis was then performed to test whether different clusters are enriched for different biological functions (Figures 3D,E). We found that the genes in cluster C2 were greatly enriched for centrosome and germ cell development GO terms (Figure 3D), raising the possibility that such genes may play a role in the proliferation and differentiation of stem cells. The genes with dynamically increased IR-containing transcript values in cluster C1 were enriched for various GO terms (Figure 3D). Furthermore, we found that the genes in cluster 3, which showed developmentally stable but relatively high IR-containing transcript values, were greatly enriched for genes associated with poly(A) RNA binding (Figure 3E). Genes in this functional category include several genes that are important for mRNA splicing, such as the pre-mRNA processing factor 3 (PRPF3) and factor 4 (PRPF4) and two non-sense mediated mRNA decay factors (SMG6 and SMG7).

### Retained Introns Display Distinct Features

**Figure 4:**
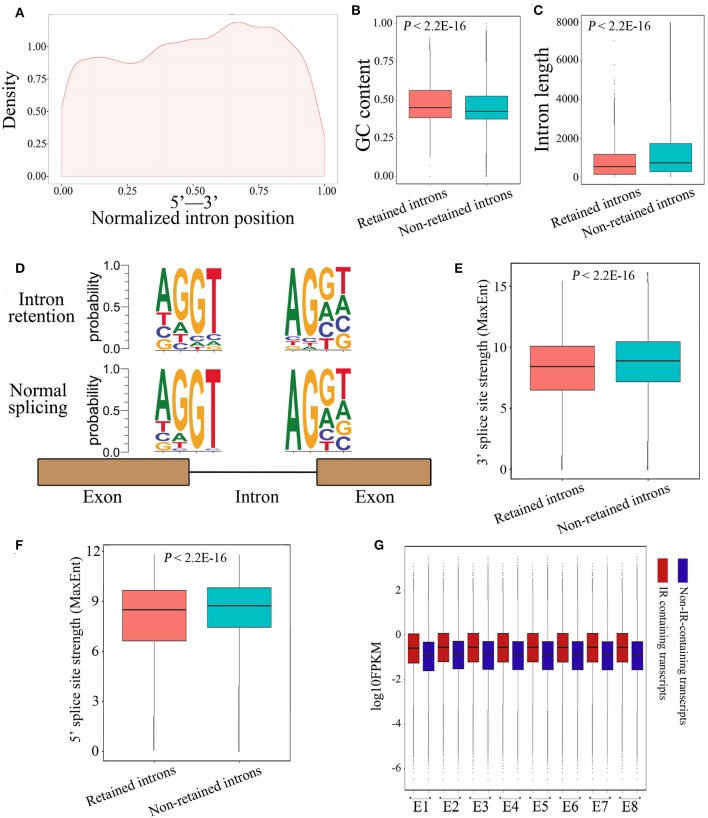
Characterization of IR events in chicken embryos identified by Iso-Seq. **(A)** Graphic representation of the location of the retained introns within the gene body. Each location was represented as the ratio of intron start location to gene length. **(B,C)** Box plots representing comparison between retained introns and non-retained introns for their length **(B)** and GC content **(C)**. **(D)** The nucleotide sequence motifs near exon-intron junctions. Four nucleotides (two exonic and two intronic) of the 5′ donor splice sites and four nucleotides of the 3′ acceptor splice sites were analyzed. **(E,F)** Box plots represent the comparison between retained introns and non-retained introns for their 3′ **(E)** and 5′ **(F)** splice site strength. The 3′ and 5′ splice-sites strengths of introns were calculated using MaxEnt (Yeo and Burge, 2004) with default parameters. **(G)** Comparison of the expression levels between IR-containing transcripts and non-IR-containing transcripts during embryonic development.

Next, we investigated whether the retained introns contained specific features not present in consistently spliced introns. Results demonstrated that retained introns tended to be localized toward the 3′ end of genes (Figure 4A). In accordance with a previous report describing differential GC content between exons and introns as a strategy for splice-site recognition (Amit et al., 2012), the retained introns in our data display slightly higher GC content (Figure 4B; 0.48 and 0.46 on average; *P* < 2.2E-16, Mann-Whitney *U* test) and are shorter in length than non-retained introns (Figure 4C; 774.6 and 1364.3 on average; *P* < 2.2E-16, Mann-Whitney *U* test). In line with the current understanding, the donor and acceptor intronic dinucleotides at splicing junctions were found to be associated with a canonical GT-AG splicing site motif (Figure 4D). As expected, the donor and acceptor intronic dinucleotides obtained at normal splicing sites were more canonical than those obtained at IR splicing junctions (Figure 4D). Meanwhile, the 3′ and 5′ splice site strength are significantly lower among the retained introns group (Figures 4E,F), suggesting that the weaker canonical dinucleotide bases at splicing junctions may be part of the mechanism underlying IR events. Moreover, we observed that the expression level of IR-containing transcripts was higher than that of non-IR-containing transcripts (Figure 4G), revealed that the IR-containing genes have highly or over transcribed pre-mRNA. It's likely that the high level of pre-mRNA coupled with introns that have weak splicing features, thus causing retention of specific introns, and in the end results the suppression of these genes. Together, these findings suggest that IR events operate through common characteristics of intronic recognition and retention, making them more susceptible to IR than other introns.

### Profiling of Global APA

**Figure 5:**
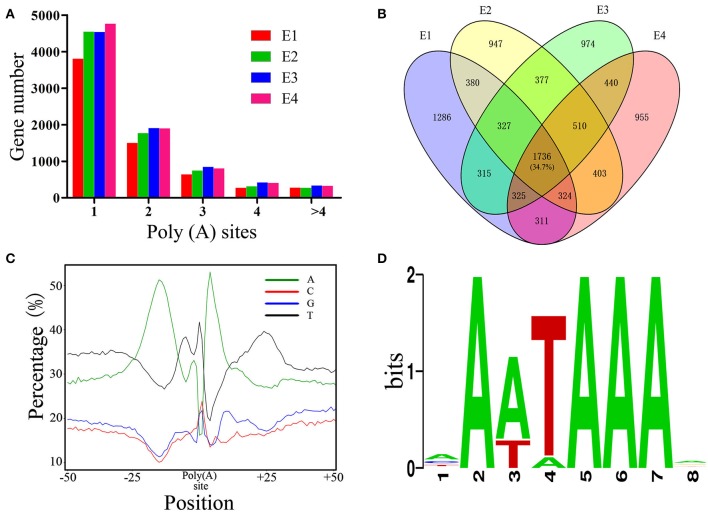
Characterization of APA in chicken embryos. **(A)** Distribution of the number of poly(A) sites per gene generated during different stages of embryo development. **(B)** Venn diagram depicting the distribution of poly(A) sites among the 5,004 genes during different stages of embryo development. Each individual cell in the diagram represents the 5,004 genes, and the overlap represents the number of genes that have the same poly(A) sites. **(C)** Nucleotide composition around poly(A) cleavage sites. **(D)** The most enriched motif in the 50 bp sequence upstream of the poly(A) tail detected by MEME.

As PacBio transcripts are full-length transcripts with a poly(A) tail, they represent an ideal dataset for the analysis of APA events (Abdel-Ghany et al., 2016). In total, 26,146 high-confidence polyadenylation sites from 10,127 genes were profiled, with an average of 2.58 polyadenylation sites aligning to each annotated gene. Of these genes, 6,082 (60.0%) had more than one polyadenylation site, suggesting that APA is a common phenomenon in the chicken transcriptome. To obtain a better understanding of the role of APA events across embryonic development, the APA distribution in PacBio data was analyzed. The results showed that the distribution of APA sites was slightly changed across the developmental stages (Figure 5A). Out of the 5,004 genes with high-confidence APA sites at all four developmental stages, only 1,736 (34.7%) genes did not change their poly(A) sites (Figure 5B), indicating that the dynamic transcriptome of the chicken embryo may also be regulated by an APA mechanism. Moreover, the nucleotide composition upstream (−50 nt) and downstream (+50 nt) of all poly(A) cleavage sites showed a clear bias toward adenine and uracil (Figure 5C). Subsequently, motifs enriched upstream of the polyadenylation sites were identified by MEME. The motif AAUAAA, which is a known canonical poly(A) signal in both plants and animals (Elkon et al., 2013; Tian and Manley, 2013), was significantly enriched (Figure 5D, *P* = 5.1E−146).

### The Majority of Transcriptomic Changes Occur During Somitogenesis

**Figure 6:**
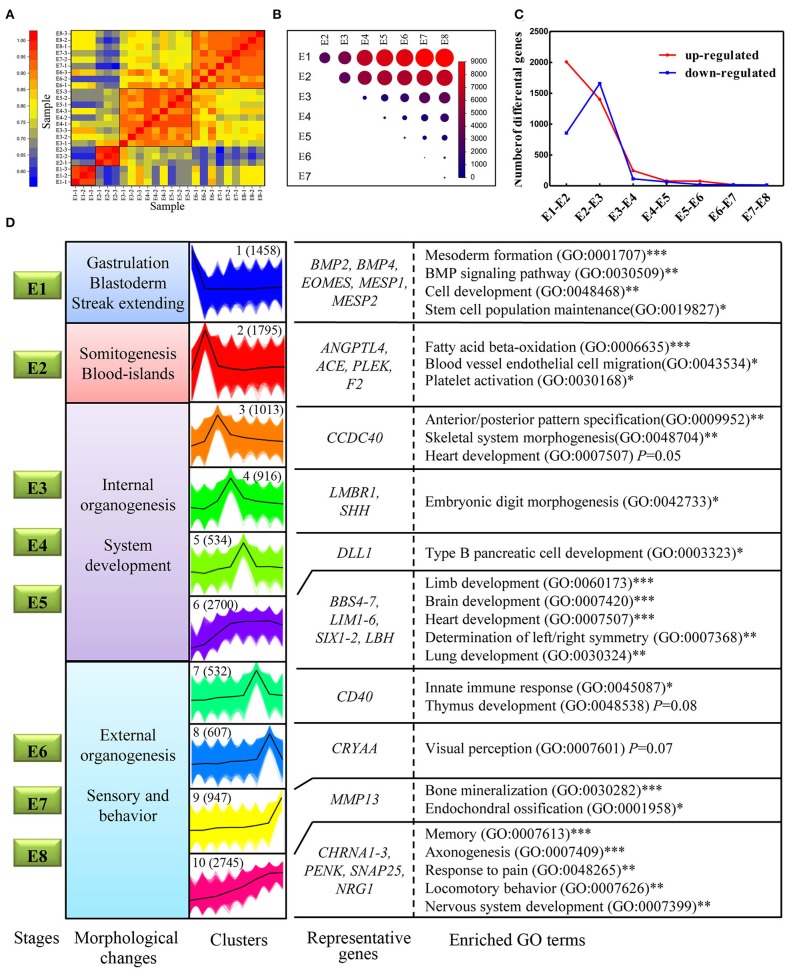
Transcriptome dynamics during chicken embryo development. **(A)** Heatmap of Pearson's correlation coefficient between transcriptomes from the eight different embryonic stages. The four clustered stages were marked with black boxes. **(B)** Visualization of the number of differentially expressed genes between each pair of stages. The size of the circles represents the number of differentially expressed genes. Genes with log2fold change > 1 and FDR < 0.01 were considered as differentially expressed genes. **(C)** The number of significantly upregulated and downregulated genes between stage transitions. **(D)** All the expressed genes were clustered into 10 k-means clusters, and distinct clusters were assigned to different morphological stages according to expression patterns. Each set of clustered genes was then subjected to GO enrichment and representative GO terms and the corresponding *P*-values are shown. ^*^ represents a *P* < 0.05; ^**^ represents a *P* < 0.01,^***^ represents a *P* < 0.001.

Spatiotemporal control of gene expression is a key to successful embryo development. Here we generated a high-resolution dynamic transcriptome representing some of the major differentiation events during embryonic development (E1–E8) using RNA-Seq. In total, we generated 228.9 million, 150-bp pair-end clean reads from the 24 established libraries (Table S3). All of the PacBio transcripts and genome-annotated transcripts were quantified using these short reads based on the PacBio GTF annotation file. A sample correlation matrix demonstrated excellent agreement among biological replicates and showed that adjacent stages are more similar to each other than temporally distant stages (Figure 6A). Moreover, this analysis also defined four developmental stages (E1, E2, E3–E5, and E6–E8) that showed distinguishable transcriptomic divergence from each other: genes identified to be differentially expressed between distant stages were much more numerous than those identified between adjacent stages (Figure 6B). Next, the dynamics of genes differentially expressed between adjacent stages was examined to investigate whether expression turnover is continuous across E1–E8. The number of significantly upregulated or downregulated genes between adjacent stages generally decreased, with the exception of a massive increase during the transition from E1–E2 to E2–E3 (Figure 6C). Strikingly, the stages of somitogenesis (E1–E3) featured the most radical transcriptomic changes, which matched the findings in studies on zebrafish and mouse embryos (Mitiku and Baker, 2007; White et al., 2017). The substantial transcriptomic dynamics from E1–E4 is in accordance with the formation of the internal organs, which demonstrates a strong correlation between morphological stages and the underlying molecular dynamics in development.

### Connections Between Gene Clusters and Biological Processes

One of the goals of this study was to explore the connection between the morphological changes and the underlying molecular changes during chicken embryo development. First, 10 distinct clusters of genes, generated by the k-means method, were distributed amongst the four developmental stages according to their expression patterns. For example, cluster I demonstrated the highest expression levels at E1 but was then rapidly downregulated. Thus, cluster I was considered to mainly function at stage E1 (Figure 6D). Subsequently, each gene cluster was analyzed for GO enrichment (only biological process-related GO terms were considered) to determine whether recognizable biological changes were obviously related to expression patterns (Figure 6D; Detailed results were shown in Table S4). Genes of cluster 1 are strongly associated with mesoderm formation (GO: 0001707) and genes in cluster 2 are associated with blood vessel endothelial cell migration (GO:0043534), which are obviously related to the morphological changes occurring at that point in development. Consistent with previous studies, some known genes essential for mesoderm formation, including *BMP2, BMP4, EOMES, MESP1*, and *MESP2*, were grouped into cluster 1. Likewise, genes known to be associated with hematopoietic functions, such as *ANGPTL4, ACE*, and *PLEK*, were grouped into cluster 2. Intriguingly, the BMP signaling pathway (GO: 0030509), which has been shown to be essential during embryogenesis, particularly for mesoderm formation and cardiac development (Winnier et al., 1995; Sm et al., 2004), was significantly enriched in cluster 1. This suggests that BMP signaling was induced and plays a critical role on the first day of incubation. Other gene sets of particular interest are clusters 6 and 10, which are both strongly associated with a range of processes involved in organogenesis and morphogenesis. However, each of these two clusters represents different specialized functions. A close inspection of the cluster 6 revealed that many known genes have a role in the development of organs, including *BBS4, LIM1, SIX11*, and *LBH*. Consistent with the observation, cluster 6 was strongly associated with the development of internal organs, such as the brain (GO: 0007420) and heart (GO:0007507). While cluster 10 was enriched in function of sensory and behavior, which relates to characteristics such as memory (GO:0007613), locomotory behavior (GO:0007626), and response to pain (GO:0048265). In this cluster, some known genes related to sensory and behaviors were observed, such as *CHRNA1-3, PENK, SNAP25*, and *NRG1*. NRG1 and SNAP25 proteins are mainly expressed in the developing nervous system, mutant mouse with deficient of these genes often display abnormalities of the nervous system (Hess et al., 1992; Pei et al., 2010). Thus, their continuous increase in expression reveals the progress of developing nervous system. Taken together, our expression dataset suggests that genes in cluster 1 may play crucial roles in gastrulation and blastoderm formation; genes in clusters 2 may primarily take part in the initiation of organogenesis; genes in cluster 3–6 are linked with internal organogenesis, whereas those in clusters 7–10 correlate well with sensory behavior. Therefore, our expression dataset defines distinct gene clusters relevant specifically to gastrulation, and to the initiation and progression of organogenesis. These findings also provide the first framework regarding the molecular basis of chicken early organogenesis.

### Identification of Novel Protein Coding Genes

**Figure 7:**
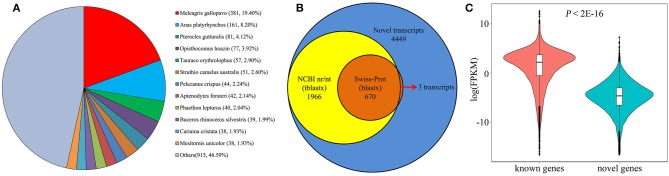
Identification of novel protein coding genes from chicken embryos. **(A)** Species distribution shows the highest match for each novel transcript in the tblastx search against NR database. **(B)** The number of identified novel transcripts (blue), and transcripts with significant sequence similarity in a tblastx search against NCBI nr/nt database (yellow) or a significant match in a blastx search against Swiss-Prot database (orange). **(C)** Comparison of mRNA expression level between known and novel protein coding genes.

Large-scale Iso-Seq analysis has enormous potential for novel gene discovery. Among the 124,559 new transcripts found in our analysis, a total of 11,471 transcripts with no overlap with any annotated genes were mapped to 6,035 novel loci. Subsequently, TransDecoder (Haas et al., 2013) was employed to identify potential protein coding transcripts, and found a total of 6,251 transcripts with multiple protein coding exons, aligned to 3,750 gene loci. To perform a comprehensive annotation, the 6,251 transcripts were then compared against NCBI non-redundant protein sequences (NR) database. From the NR analysis, 1,802 transcripts were annotated as known chicken transcripts. The rest of 4,449 transcripts produced by 2,686 gene loci, hence are likely novel genes. We next assessed the presence of these novel genes in other organisms using tblastx and blastx tools. As a result, a total of 1,966 (44.19%) transcripts have at least one significant hit in the tblastx search against the nucleotide collection (nr/nt) database (Figure 7A). And 670 (15.1%) transcripts have significant hits in the blastx search against the Swiss Prot proteins (SwissProt) database (Figure 7B). Interestingly, most of the significant hits were against *Aves*, the next is *Xenopus laevis* and *Danio rerio* sequence. Only 260 (13.2%) transcripts have a match against mammal sequence, indicating that these novel genes are less conserved during the evolution. Moreover, we found that all but 3 of these 670 transcripts also have significant hits in the tblastx search (Figure 7B). Given the high confidence of proteins in SwissProt, makes it convincible that these novel transcripts are transcribed from the identified novel gene loci. For the remaining 2,483 transcripts without blast hits, it's probably due to two reasons. First, some of the genes may be chicken specific. Second, low conservation among organisms may make some transcripts escape from the blast search. Analysis of FPKM values of the 1,330 potentially novel genes showed that while 222 (16.7%) of these genes were expressed at a relatively low level (FPKM <0.1 in all samples), the majority were readily detectable in our analysis. Five novel genes were randomly selected and validated by RT-PCR (Figure S3), demonstrating that these represented bona fide loci. Overall, we found that the novel genes, on average, were expressed at lower levels compared with known protein coding genes (Figure 7C; *P* < 2.2E−16, Mann-Whitney *U* test), possibly explaining why these transcripts were not identified in previous studies.

## Discussion

Chickens have unique attributes for a vertebrate model that allow detailed study of developmental biology. The transcriptome profiling of chicken embryos creates an opportunity to advance our understanding of the molecular regulation of embryo development. Here, we focused on early embryo development due to its diverse transcriptional repertoire. The initial 25 h of chicken embryonic development from a zygote to a blastoderm occurs in the oviduct. After oviposition, the embryonic gastrulation and then organogenesis all take place *in vitro*. During the first 1–3 days of incubation, the progress of somitogenesis is prominent. Subsequently, rapid changes in visceral arches occur, and the primary structure of many organs is distinguishable after nine days of incubation. Therefore, E1–E8 is a critical period for the study of developmental biology. Previously, a study provided transcriptomic data for chicken embryo, revealing an hourglass-like divergence in the embryogenesis of the soft-shell turtle and the chicken (Wang et al., 2013). A recent work explored the entire transcriptome of pre-oviposited early chicken embryos using single and bulked embryonic RNA-Seq (Hwang et al., 2018a,b). It was the first whole transcriptomic exploration of pre-oviposited early chicken embryos and provided a valuable resource for investigating early embryogenesis. Here, we present a valuable resource for investigating gastrulation, somitogenesis, and organogenesis of the chicken embryo. However, these analyses used whole embryo samples, which is a strategy that is not suitable for investigating postembryonic development. Recently, a study revealed the intricate patterns of gene expression along the primitive streak of chicken embryo by ordering single-cell transcriptomes (Vermillion et al., 2018). With this in mind, we believe that identifying tissue-resolved or even single-cell-resolved (Briggs et al., 2018; Wagner et al., 2018) transcriptional landscapes will aid in the understanding the postembryonic development.

In agreement with previous reports (Kuo et al., 2017), our data further emphasizes the complexity of the chicken transcriptome. We identified 96,581 new, previously unannotated isoforms of previously known gene loci in the chicken genome. The newly annotated transcripts represent about a 3-fold increase in identified transcripts. Moreover, we discovered more than 3,000 novel gene loci, and these novel loci were validated using short-read sequencing data, homology sequences, and RT-PCR. The discovery of a large number of new transcripts benefits from the application of new technologies. Likewise, there are more than 160,000 annotated transcripts corresponding to 22,734 protein-coding genes in the latest human genome annotation, built on short-read and long-read sequencing. Thus, the transcriptomic complexity of a chicken is similar to that of a human. It should be noted that capturing mature, stable, and functional mRNA molecules is still significantly challenging. For instance, non-sense-mediated decay products, pre-mRNAs, not fully degraded mRNA, and fragmentary mRNA may also be captured when preparing sequencing libraries. Therefore, more exact and effective strategies are needed for preparing sequencing libraries.

AS is a major contributor in increasing diversity and transcriptomic complexity (Vanichkina et al., 2017). To shed light on this issue, we applied an integrated approach using Iso-Seq and RNA-Seq to characterize the AS events. Here, we show that the AS landscape varies with organogenesis, with a marked increase in the most abundant AS event, IR, during embryonic development, suggesting IR as a widespread regulatory mechanism during this developmental period (Figure 2). We have also showed that retained introns have distinct features (i.e., short length, high GC content), and demonstrated that both dynamic and stable IR-containing transcripts correlate with distinct functional gene classes. Taken together, our study reveals that orchestrated IR events play an expanded regulatory role in chicken embryonic development.

Chicken embryogenesis has long been established based on the morphological changes that occur during development ted (Hamburger and Hamilton, 1951; Watt et al., 1993). Many morphological observations, advanced electron microscopy technology and monoclonal antibodies have been applied to generate anatomical descriptions at the ultrastructural and molecular levels. However, these studies led to few new insights into molecular mechanisms of development. Especially, an understanding of the temporal control of gene expression during embryonic development has thus far been absent. Here, large-scale transcriptome analysis of the critical developmental period provides a temporal profile of gene expression. Subsequently, genes were clustered into groups according to their temporal profiles by using the k-means clustering method. Such clustering facilitates the functional exploration of each gene cluster, and allows for greater interpretive potential when associated with various developmental stages. For example, the clusters of genes (cluster 3–6), whose expression mainly changed in the internal organogenesis period, are strongly enriched during internal organ development such as that observed in the heart and lungs. The integrated analysis between the dynamic nature of early embryonic biological processes and functional gene clusters provides insight into chicken embryonic development. Moreover, we were able to describe the development of the sensory and central nervous systems, which can hardly be described by morphological changes. Overall, this study provides the first insights into the temporal control of genes expressed during early embryonic development, although the connections between gene clusters and biological processes remain to be explored in detail.

In conclusion, we explored the intricacies of embryonic development at the molecular level, by obtaining insight into the high-resolution time course of mRNA expression. We preliminarily characterized the developmental milestones by sample correlation matrix analysis and defined clusters of genes that have distinct functions, including in mesoderm formation, internal organ development, and external sensory organ development. Our findings provide a powerful resource for increasing our understanding of the developmental biology of the chicken embryo. It is likely that in the future, both this resource and our understanding of development will be further expanded by the use of large-scale single-cell RNA sequencing technology (Briggs et al., 2018; Wagner et al., 2018).

## Data Availability

All data generated in this manuscript have been deposited in NCBI under the accession number PRJNA488330.

## Ethics Statement

The animal study was reviewed and approved by Animal Welfare Committee of China Agricultural University.

## Author Contributions

NY conceived the study, participated in the experiment design, critical discussion, and manuscript preparation. JR performed the experiments, carried out the functional analysis, and drafted the manuscript. CS contributed to the experiment design, part of data analysis, and manuscript preparation. MC participated in the data analysis, critical discussion, and manuscript preparation.

### Conflict of Interest Statement

The authors declare that the research was conducted in the absence of any commercial or financial relationships that could be construed as a potential conflict of interest.

## Supplementary Material

Figure S1 CIRCOS visualization of different data at the genome-wide level. **(A)** Karyotype of the chicken genome. **(B)** The abundance of FLNC transcripts in physical bins of 1 Mb for each chromosome. **(C)** The abundance of short reads generated by RNA-Seq in physical bins of 1 Mb for each chromosome. **(D)** The density of isoforms generated by Iso-Seq. Isoform density was calculated in a 1 Mb sliding window at 20 kb intervals. **(E)** The density of known isoforms generated by Ensembl database. Isoforms density was calculated in a 1 Mb sliding window at 20 kb intervals. **(F)** Linkage of fusion transcripts: red, intra-chromosomal; green, inter-chromosomal.

Figure S2 RT-PCR validation of AS events during chicken embryo development.

Figure S3 RT–PCR validation of novel genes.

Table S1 Primers sequences used for RT-PCR (DOCX 18 kb).

Table S2 Summary of data generated by Iso-Seq (XLS 14 kb).

Table S3 Summary of data generated by RNA-Seq (XLS 14 kb).

Table S4 List of enriched GO terms for different gene clusters (XLS 83 kb).
