# Supplementary figures and images for: Dynamic Transcriptional Landscape of the Early Chick Embryo
Source: Front Cell Dev Biol. 2019 Sep 12;7:196. doi: 10.3389/fcell.2019.00196 (PMC6751280; doi:10.3389/fcell.2019.00196)

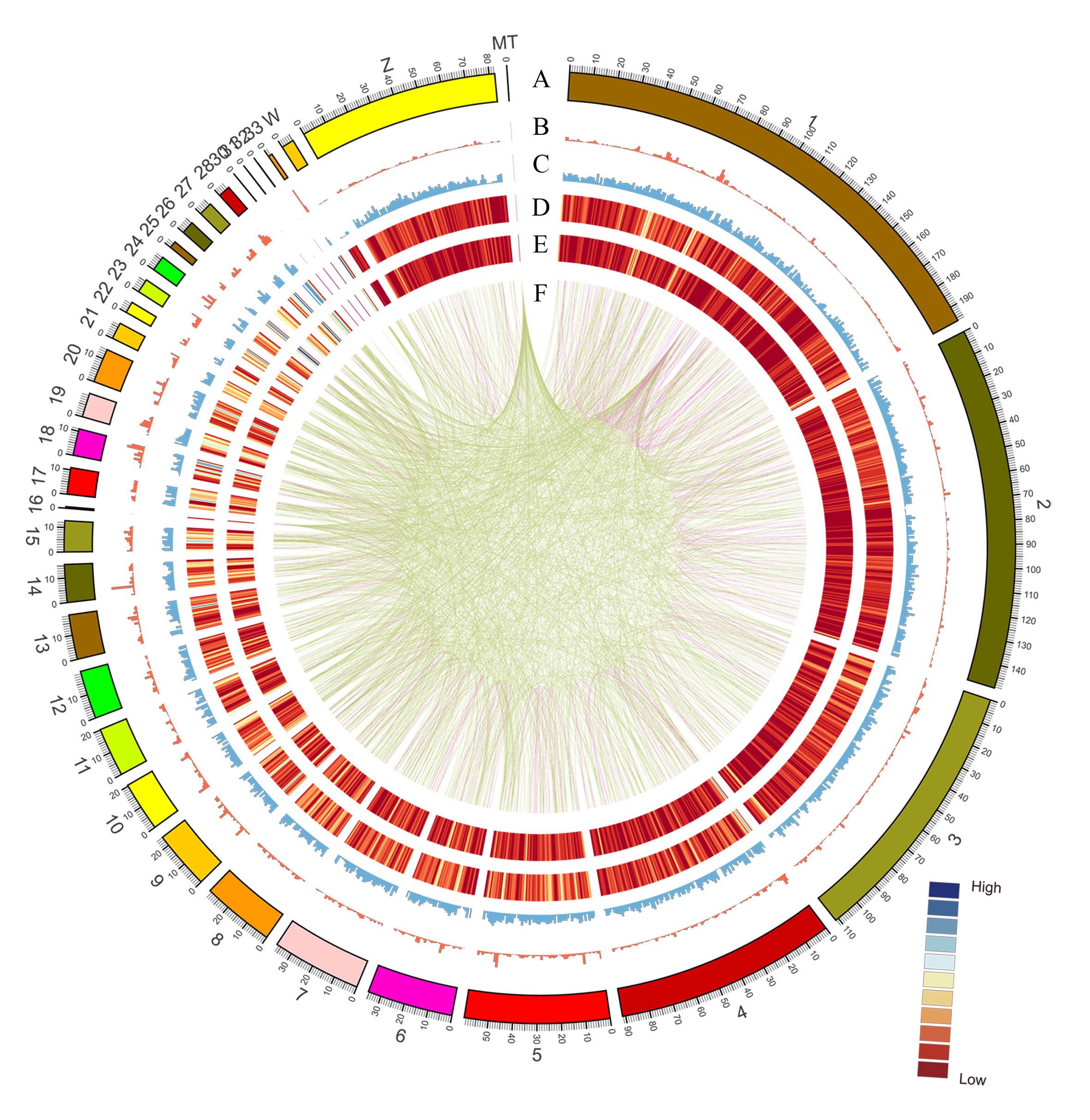

Supplement: Figure S1 — CIRCOS visualization of different data at the genome-wide level. (A) Karyotype of the chicken genome. (B) The abundance of FLNC transcripts in physical bins of 1 Mb for each chromosome. (C) The abundance of short reads generated by RNA-Seq in physical bins of 1 Mb for each chromosome. (D) The density of isoforms generated by Iso-Seq. Isoform density was calculated in a 1 Mb sliding window at 20 kb intervals. (E) The density of known isoforms generated by Ensembl database. Isoforms density was calculated in a 1 Mb sliding window at 20 kb intervals. (F) Linkage of fusion transcripts: red, intra-chromosomal; green, inter-chromosomal. [file Image_1.JPEG]

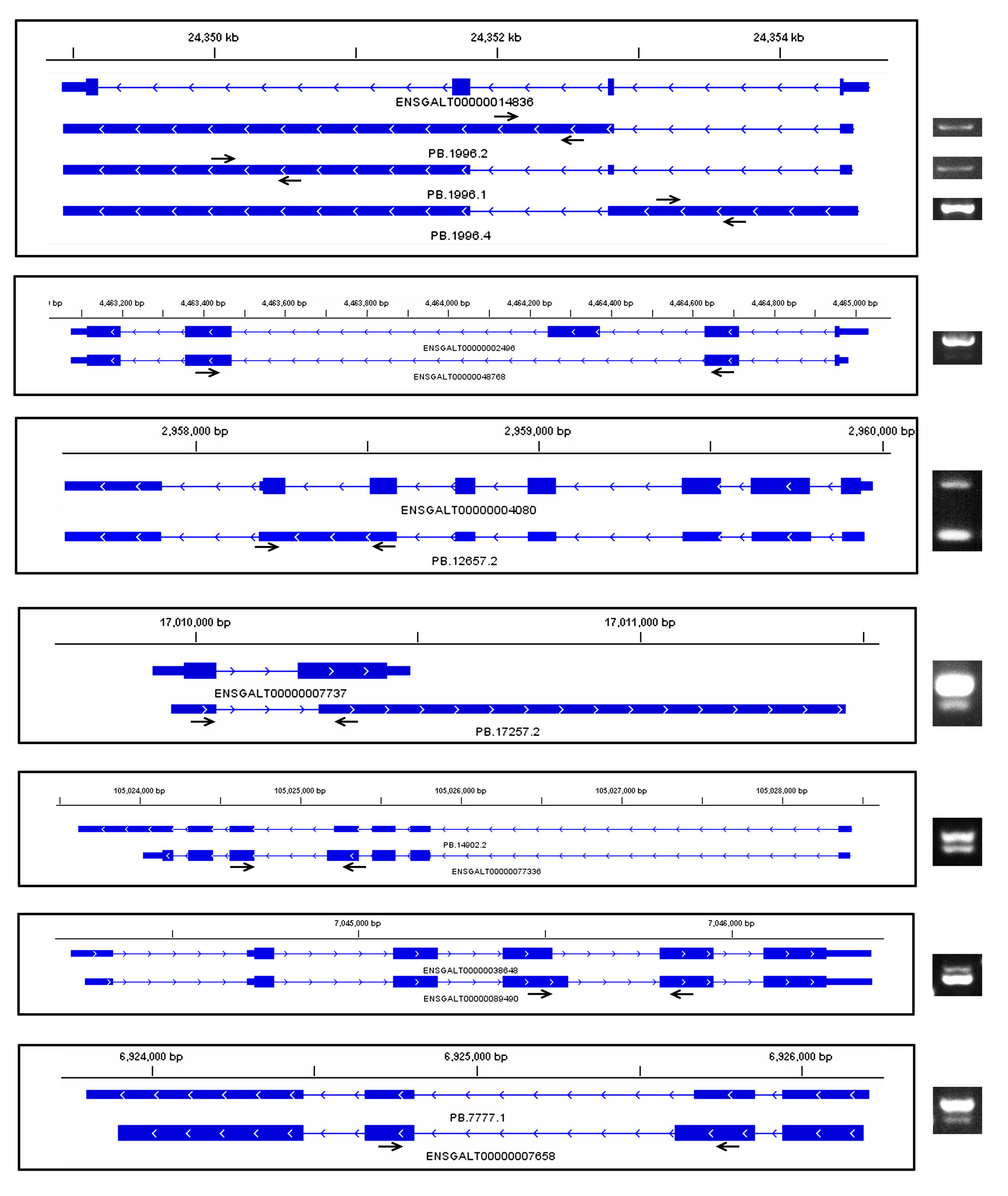

Supplement: Figure S2 — RT-PCR validation of AS events during chicken embryo development. [file Image_2.jpg]

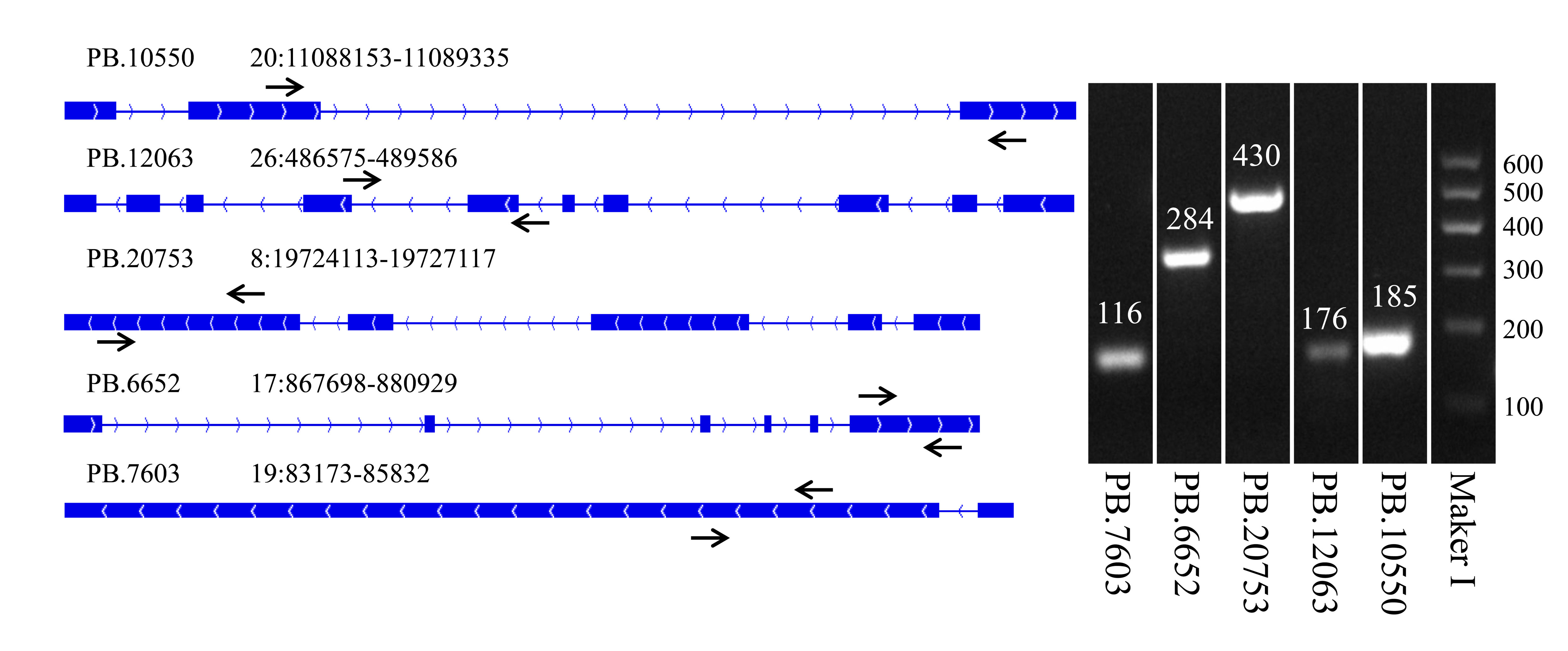

Supplement: Figure S3 — RT–PCR validation of novel genes. [file Image_3.JPEG]
