# Supplementary material for: Dynamic Transcriptional Landscape of the Early Chick Embryo
Source: Front Cell Dev Biol. 2019 Sep 12;7:196. doi: 10.3389/fcell.2019.00196 (PMC6751280; doi:10.3389/fcell.2019.00196)
Supplement: Table S1 — Primers sequences used for RT-PCR (DOCX 18 kb). [file Table_1.DOCX]

Table s1 Primer sequences used for RT-PCR

| Transcript ID | primers |
| --- | --- |
| PB.1996.1 | F:GCAGACCTCAAAAACGGCAG  R:ACTGCAGCCCTCTGAAAGTC |
| PB.1996.2 | F: GGTGAGACGTTAGTGCTGCT  R: TGAGGGTTCACACTAACGGC |
| PB.1996.4 | F: TGGTATCCTGGGCTCCATCA  R: GGTTGGAAGGGACCTCAAGG |
| ENSGALT00000048768  ENSGALT00000002496 | F: CGTGGGAGCCGTGATTAACT  R: AGATCTGCACACTCCTTGGC |
| ENSGALT00000004080  PB.12657.2 | F: CAGTCCCTGGTCGATATGGC  R: GCTGTTGGTGGATGGGAAGA |
| ENSGALT00000007737  PB.17257.2 | F: CTGGAGGTGGACATGATGGC  R:CTGCTGCATGCGCTTGAG |
| PB.14902.2  ENSGALT00000077336 | F:GAAGGAATACAACCCGCTGC  R:GGTTGAGCTCCAGGATCTGT |
| ENSGALT00000038648  ENSGALT00000089490 | F:CCTCCCTGAGACCCATCAAA  R:GAGCTGTCTTTGTAGTCGCC |
| ENSGALT00000007658  PB.7777.1 | F:ACCATCGCTAAGTCACGGAG  R:GATGAGGACTATGGGCAGCT |
| PB.10550 | F:ATCTGCGCTCAGGTTGTCAG  R:CTTGAGCTTGCCTACGGCTT |
| PB.12063 | F:ATGAGAGCTTCGTCAGTGCC  R:GAATGAGTCGGAACGCCTGA |
| PB.20753 | F: TACATTCAGCCCAGTGAGCA  R: GAGTGACAACAGAGTGGGGA |
| PB.6652 | F: TCCTTCCCATGCAGTCCAAT  R: TCTCAGGAGTGTGCAAGGAG |
| PB.7603 | F: CTCTGACATCTCCACTGGCA  R: GAACACATGCAGCACACAGA |
|  |  |
